# Effects of Sublethal Fungicides on Mutation Rates and Genomic Variation in Fungal Plant Pathogen, *Sclerotinia sclerotiorum*

**DOI:** 10.1371/journal.pone.0168079

**Published:** 2016-12-13

**Authors:** B. Sajeewa Amaradasa, Sydney E. Everhart

**Affiliations:** Department of Plant Pathology, University of Nebraska-Lincoln, Lincoln, Nebraska, United States of America; Beijing Institute of Microbiology and Epidemiology, CHINA

## Abstract

Pathogen exposure to sublethal doses of fungicides may result in mutations that may represent an important and largely overlooked mechanism of introducing new genetic variation into strictly clonal populations, including acquisition of fungicide resistance. We tested this hypothesis using the clonal plant pathogen, *Sclerotinia sclerotiorum*. Nine susceptible isolates were exposed independently to five commercial fungicides with different modes of action: boscalid (respiration inhibitor), iprodione (unclear mode of action), thiophanate methyl (inhibition of microtubulin synthesis) and azoxystrobin and pyraclostrobin (quinone outside inhibitors). Mycelium of each isolate was inoculated onto a fungicide gradient and sub-cultured from the 50–100% inhibition zone for 12 generations and experiment repeated. Mutational changes were assessed for all isolates at six neutral microsatellite (SSR) loci and for a subset of isolates using amplified fragment length polymorphisms (AFLPs). SSR analysis showed 12 of 85 fungicide-exposed isolates had a total of 127 stepwise mutations with 42 insertions and 85 deletions. Most stepwise deletions were in iprodione- and azoxystrobin-exposed isolates (n = 40/85 each). Estimated mutation rates were 1.7 to 60-fold higher for mutated loci compared to that expected under neutral conditions. AFLP genotyping of 33 isolates (16 non-exposed control and 17 fungicide exposed) generated 602 polymorphic alleles. Cluster analysis with principal coordinate analysis (PCoA) and discriminant analysis of principal components (DAPC) identified fungicide-exposed isolates as a distinct group from non-exposed control isolates (PhiPT = 0.15, *P* = 0.001). Dendrograms based on neighbor-joining also supported allelic variation associated with fungicide-exposure. Fungicide sensitivity of isolates measured throughout both experiments did not show consistent trends. For example, eight isolates exposed to boscalid had higher EC_50_ values at the end of the experiment, and when repeated, only one isolate had higher EC_50_ while most isolates showed no difference. Results of this support the hypothesis that sublethal fungicide stress increases mutation rates in a largely clonal plant pathogen under *in vitro* conditions. Collectively, this work will aid our understanding how non-lethal fungicide exposure may affect genomic variation, which may be an important mechanism of novel trait emergence, adaptation, and evolution for clonal organisms.

## Introduction

Fungicide resistance in populations of fungal plant pathogens has three phases: emergence, selection, and adjustment [1]. Emergence involves generation of a resistance strain via mutation, also called acquired resistance [2], and the selection phase results in an increase of the proportion that are resistant in the population. Once the resistant population has reached an intermediate level in the adjustment phase, a change in fungicide dose or mode of action is needed in order to achieve disease control. Most research data are available for the selection phase. For example, traditional fungicide efficacy studies identify fungicide resistant strains within populations, which may represent preexisting rare mutations [3]. There is little to no published data to characterize the emergence phase for fungal plant pathogens. Research on bacterial pathogens of humans and livestock animals suggests sublethal antibiotic exposure plays an important role in antibiotic resistance emergence via horizontal gene transfer, recombination, and both random and non-random mutations [4,5]. In fungi, antifungal drug-induced chromosomal changes have been reported in human fungal pathogens such as *Candida albicans* [6] and *Cryptococcus neoformans* [7]. Thus, a clear picture is emerging that exposure to sublethal fungicides may increase mutation rates, a pre-requisite to resistance emergence, and may also serve as heretofore unexplored source of population genetic variation of importance for primarily clonal fungal plant pathogens.

Fungal plant pathogen exposure to sublethal doses of fungicides may occur for fungicides applied in an agricultural setting. In some cases, farmers intentionally reduce fungicide dose, reduce the number of fungicide applications, or delay application until disease symptoms are visible. In other cases, sublethal exposure may be caused by incomplete plant canopy penetration or dilution within plant tissues. Indirect mechanisms may also exist, including waterways contaminated with pesticides washed off from upland agricultural areas that could create sublethal conditions for plant pathogens in irrigated fields downstream [4] and environmental conditions that degrade chemicals *in situ*, resulting in uneven or reduced rates.

A few studies have characterized the effects of sublethal fungicides on fungal plant pathogens *in vitro*. For example, isothiocyanates (ITCs) are natural defense-related compounds with antifungal properties and exploited as soil biofumigants. ITCs are derived from the precursor glucosinolate, a secondary metabolite synthesized by *Brassicas*. Since the mode of action of ITCs is unknown, effects of exposure were tested *in vitro* on *Alternaria alternata* to determine if resistance would develop [8]. Isolates from tomato and cabbage were exposed to low concentrations of two forms of ITC, allyl-isothiocyanate (AITC) and benzyl isothiocyanate (BITC), in gradual increments until isolates were able to grow under selective pressure. Molecular differences between non-exposed and ITC-exposed isolates were characterized using genotyping at five inter simple sequence repeats (ISSR). BITC-adapted isolates had more than twice as many polymorphisms (118) compared to AITC-adapted isolates (51), where isolates from cabbage had more polymorphisms (116) compared to isolates from tomato (53). The authors concluded both chemical composition of ITC and adaptation on the host plant influenced the number of mutations *in vitro*. In a similar experiment, continuous exposure to sublethal doses of a demethylation inhibitor (DMI; SYP-Z048) and a quinone outside inhibitor (QoI; azoxystrobin) fungicide were tested individually and in mixture treatments on *Monilinia fructicola* mycelium of four isolates [9,10]. Mutagenesis was assessed using 15 microsatellite (SSR) loci and transposition of *Mftc1*, a multicopy transposable element in *M*. *fructicola*. Their analysis showed mutagenesis at 8 SSR loci in one isolate exposed to azoxystrobin and movement of *Mftc1*, which targets the upstream promoter region of MfCYP51, in isolates exposed to azoxystrobin (alone and mixed with SYP-Z048). Finally, contrary to studies with *A*. *alternata* and *M*. *fructicola*, a study carried out to assess stability of SSR loci in plant pathogen, *Botrytis cinerea*, showed growth for 20 transfers with increasing concentration of antifungal antibiotic pyrrolnitrin and fungicide iprodione did not result in mutation at nine SSR loci [11].

Results of the previous studies above suggest that more research is needed to ascertain the affect of sublethal fungicides on mutation rates and new trait emergence. Although several authors have speculated that plant pathogenic fungi exposed to environmental stress and/or sublethal fungicide doses may increase mutation rates and lead to resistance [12,13], no previous studies have rigorously tested this hypothesis or used a genome-wide approach. Thus, the goal of our current work was to elucidate the impact of sublethal fungicide exposure on genomic variation, mutation rates, and fungicide sensitivity, using a model plant pathogen with a reference genome to enable subsequent whole-genome studies. In addition to the role of mutations in fungicide resistance emergence, such mutations may be a more important source of genetic variation in haploid genome evolution, as compared to diploid or polyploid genomes where mutations can be more complex and phenotype expression delayed [2]. Consequently, we sought to test our hypothesis using a fungal plant pathogen with a haploid genome, high genomic stability, and low population genetic variation. One such model organism is *Sclerotinia sclerotiorum*, which is primarily homothallic and where multiple studies have found little evidence of sexual out-crossing [14–17]. This pathogen is a necrotrophic fungus [18] with a host range of more than 400 plant species in 75 families [19]. Sclerotia can survive in soil for years and produce apothecia in favorable conditions. Since most crops are either susceptible or partially resistant to this pathogen, fungicide applications are recommended for disease management [18,20].

*Sclerotinia sclerotiorum* has a fully annotated and sequenced genome, with little evidence of genomic plasticity. For example, it is known that some filamentous plant pathogens have large genomes with high plasticity in repeat-rich, gene sparse or isochore-like regions, facilitated by TE activity. Classic examples include obligate biotroph and hemibiotroph, *Blumeria gramminis* and *Phytophthora infestans*, which have genomes of 120 Mbp and 240 Mbp, with TE content exceeding 50% [21]. In comparison, *S*. *sclerotiorum* has a small haploid genome of 38 Mbp, where TEs comprise 7% of the genome [22]. In addition, a previous study maintained continuous vegetative growth of *S*. *sclerotiorum* for one year and showed no variation in seven SSR loci and 56 AFLP alleles [23]. Collectively, these results suggest that *S*. *sclerotiorum* has a stable genome with few endogenous mechanisms for adaptation, thus resulting in greater dependence on exogenously induced genomic mutations (not mediated by TE activity) for adaptive evolution and survivability under environmental stress.

Fungicides have different modes of action on fungal metabolism and growth, which may have different effects in sublethal concentrations. To rigorously test our hypothesis, we selected five commercial fungicide formulations that have different modes of action for the present study: thiophanate methyl, azoxystrobin, pyraclostrobin, iprodione, and boscalid. Thiophanate methyl is a benzimidazole class of fungicide, with a mode of action that inhibits assembly of microtubules, preventing nuclear division of fungal cells [24]. Both azoxystrobin and pyraclostrobin are classified as strobilurin fungicides that have a mode of action to inhibit the mitochondrial electron transfer chain and disrupt metabolic activity that requires ATP [25]. Iprodione is classified as a dicarboximide fungicide with an unknown mode of action [26]. Boscalid is classified as a succinate dehydrogenase inhibitor (SDHI) fungicide that has a mode of action to target succinate dehydrogenase complex in the respiration chain and inhibits fungal respiration by blocking ubiquinone-binding sites in mitochondrial complex II [27].

To assess genomic variation before and after fungicide exposure, isolates were genotyped at six simple sequence repeat (SSR) loci [28] and using three amplified fragment length polymorphic (AFLP) markers. These complementary genotyping techniques were selected due to differences in expected mutation rates, mechanisms of mutations captured, and portion of the genome represented by each marker. SSRs are short tandem repeats, typically 2–6 bp in length, and used extensively for population genetic studies due to high polymorphism, codominant and quantitative nature, and assumed to represent non-coding regions that lack selection [28–30]. Polymorphism in fragment length at SSR loci is thought to arise via polymerase slippage that add or delete a tandem repeat in stepwise fashion [30]. Complementary to SSRs, AFLP is a multilocus marker with ability to amplify 50–100 loci in the genome [31,32]. AFLP is a dominant marker that utilizes restriction sites to generate polymorphic DNA fragments up to 500-bp in length, which can vary due to insertions and deletions or single nucleotide polymorphism (SNP) mutations at the restriction site [32]. AFLP regions are amplified throughout the genome, with the majority in non-coding regions [33,34], making them appropriate to use in population studies.

The objectives of the present study were to: (i) assess genomic variation of *S*. *sclerotiorum* isolates exposed to long-term sublethal doses of fungicides *in vitro* using SSR and AFLP markers, (ii) estimate the affect of sublethal stress on mutation rates at SSR loci, and (iii) determine *in vitro* trends of effective concentration of fungicides required for 50% growth inhibition (EC_50_) over time in *S*. *sclerotiorum* isolates exposed to sublethal doses of fungicides.

## Materials and Methods

### Isolates and Experimental Design

Nine fungicide-sensitive *S*. *sclerotiorum* isolates (ID 152, 462, 467, 555, 587, 588, 594, 646, and 655) were selected from 366 isolates genotyped and phenotyped previously [35]. Isolates were collected 1980 to 2005 from dry bean (*Phaseolus vulgaris*) in seven states (CA, CO, MN, ND, NE, OR, WA; Table 1). Host cultivars were ‘Bunsi’, ‘Beryl’, ‘Pinto’, or ‘Great northern’, which were field grown without fungicide applications. A single sclerotium of each isolate stored in the culture collection of J. Steadman (University of Nebraska) was plated onto water agar and hyphal tips from the leading edge of the growing culture were used to initiate each isolate in this study. Multilocus genotypes generated previously were used to verify that each isolate was composed of a single homokaryotic genetic profile [35].

**Table 1:** Isolates of *Sclerotinia sclerotiorum* used in the present experiment.

Isolate ID	Origin	Year collected	Aggressiveness^a^	MCG^b^	Host cultivar^c^
152	Nebraska	1980	3.9	4	Great Northern
462	Washington	2003	4.6	57	Bunsi
467	Colorado	1996	4.6	45	Pinto
555	Minnesota	2004	6.4	44	Bunsi
587	Oregon	2004	5.5	5	Beryl
588	Oregon	2004	5.3	4	Beryl
594	California	2004	4.6	21	Bunsi
646	Washington	2005	5.4	60	Bunsi
655	North Dakota	2005	4.0	46	Bunsi

^a^ Aggressiveness was rated on scale of 1–9 using the straw test method with increasing numbers representing higher aggressiveness [36].

^b^ MCG: Mycelial compatibility group.

^c^ Common bean (*Phaseolus vulgaris*) cultivar.

Each of the nine selected *S*. *sclerotiorum* isolates were independently grown on a fungicide gradient and successively sub-cultured from the 50–100% growth inhibition zone for a total of 12 times (described below). This was performed for a total of five commercial fungicides labeled for white mold control, representing four modes of action: respiration inhibitor boscalid (Emerald, 70% a.i. BASF Corporation, Research Triangle Park, NC), iprodione (26GT, 23.3% a.i., Bayer Crop Science LP, Montvale, NJ) with unclear mode of action, microtubulin synthesis inhibitor thiophanate methyl (T-STORM 50WSB, 50% a.i., LESCO, Inc., Cleveland, OH), and quinone outside inhibitors (QoI) azoxystrobin (Heritage, 8.8% a.i., Syngenta Crop Protection, Greensboro, NC) and pyraclostrobin (Insignia SC, 23.3% a.i., BASF). Negative controls were included for each isolate, which were simultaneously subjected to serial transfers, with growth on PDA lacking fungicides. The entire experiment was repeated. Since a single generation required four days to complete, each experiment was continuously carried out for 48 days. Isolates were genotyped using 6 SSR loci, following which, half of the fungicide-exposed isolates showing mutation at any SSR locus and their corresponding control isolates were preferentially selected for AFLP genotyping. This method of selection was used because SSR loci are considered highly variable [30] and thus were expected to be an indicator of mutational change.

Each isolate was given a unique designation with isolate ID number, experiment, and treatment. To distinguish isolates from the first and second experiment, ‘Exp1’ and ‘Exp2’ were used. Each of the nine progenitor isolates was given the ‘G0’ designation and negative control isolates at the end of the experiment were given the ‘G12’ designation and control denoted as ‘Con’. Since each of the nine G0 isolates was the progenitor to a fungicide-exposed isolate at G12, it was unnecessary to give fungicide-exposed isolates the G12 designation. Each fungicide-exposed isolate was given a designation corresponding to the fungicide it was exposed to, with each abbreviated as follows: ‘TM’ (thiophanate methyl), ‘Bos’ (boscalid), ‘Ip’ (iprodione), ‘Az’ (azoxystrobin), and ‘Py’ (pyraclostrobin).

### Fungicide Gradient

The Autoplate 5000 (Advanced Instruments, Inc. Norwood, MA) was used to deposit a stock solution of each fungicide onto an oversized (150 mm diameter), rotating Petri plate containing solid media. This instrument has a 1 mm diameter stylus that ejects solution at a rate that decreases as applied from center to periphery of the Petri plate, in a spiral pattern. Plates are allowed to stand 2–4 hours prior to inoculation, which allowed permeation of solution into media. This instrument applied fungicides with high accuracy and precision, allowing estimation of fungicide concentration at a radial distance up to 0.5 mm increments.

Optimal fungicide deposition mode and starting stock solution concentration was determined prior to beginning the experiment. Each fungicide stock solution was deposited onto solidified PDA in oversized Petri plates, prepared by adding 50 ml of PDA and stored at least 48 hours at room temperature to obtain a dry surface and no condensation prior to fungicide application. Fungicide deposition was made using either the exponential or proportional mode, where the exponential mode creates a fungicide gradient from center to periphery that is approximately to 300:1 and the proportional mode is 3:1. The ‘exponential’ mode of deposition was suitable for most fungicides in the present study. In the case of iprodione, the ‘proportional’ mode of deposition was used because the ‘exponential’ mode resulted in a short growth-to-no growth interphase that hindered accurate estimation of 50% growth inhibition. Fungicide concentrations used to feed the dispensing stylus and actual deposited concentration at the center and periphery of plates are given in Table 2. Each fungicide concentration was optimized to avoid either too little growth or overlapping mycelial growth of adjacent replicates. PDA media used for QoI fungicide deposition were amended with 70 ppm salicylhydroxamic acid (SHAM) dissolved in acetone. SHAM was added to inhibit the alternative respiration pathways used by fungi *in vitro* to avoid fungicide toxicity and has been previously evaluated for *S*. *sclerotiorum* [37]. Fungicide-deposited Petri plates were set in an air recirculating biosafety cabinet for 2–4 hours to absorb chemicals before inoculating with *S*. *sclerotiorum* isolates; plates more than 4 hours old were not used.

**Table 2:** Concentration (ppm) of fungicide stock solutions and resultant concentration gradient when deposited^a^ onto a 150 mm PDA plate in a concentric spiral pattern.

Fungicide	Stock	Center	Peripheral
Boscalid	750	9.07	0.05
Iprodione	400	2.58	0.807
Thiophanate methyl	9000	108.79	0.57
Azoxystrobin	250	2.87	0.01
Pyraclostrobin	200	2.34	0.01

^a^ Exponential mode of deposition used for all fungicides except iprodione (proportional mode).

### Sublethal Fungicide Exposure

Fungicide-deposited plates were inoculated with a uniform distribution of *S*. *sclerotiorum* using mycelium pre-grown on 50 x 4 mm sterile filter paper strips placed perpendicular to the fungicide gradient, as described previously [38]. To generate these pre-inoculated strips, each *S*. *sclerotiorum* isolate was grown on a 100 mm diameter Petri plate and aerial hyphae harvested (*S*. *sclerotiorum* does not produce conidia in culture) before mycelial growth reached the edge of the plate. Harvested mycelium was added to 500 μl of sterile water in a 2 ml microtube with two sterile glass beads and homogenized for 20 seconds using a fixed-speed Fastprep homogenizer (MP Biomedical, Solon, OH). Filter strips (50 x 4 mm) were made from Whatman grade 1 paper (GE Healthcare Bio-Sciences, Pittsburg, PA) pre-cut using a mechanical paper shredder and scissors, prior to sterilization. Filter strips were aseptically pressed onto PDA plates and 45 μl of mycelial homogenate applied. Inoculated strips were incubated for 40 hours at 25°C to allow mycelial colonization of paper and transferred to fungicide deposited plates.

Four strips (two isolates per plate) were placed perpendicular to the fungicide gradient in each Petri plate. Simultaneously, PDA in 100 mm diameter Petri plates (or PDA amended with 70 ppm SHAM for comparison to QoI fungicide-deposited Petri plates) were used as a negative control for each isolate, with a single inoculated filter strip placed near the periphery. Negative controls (also used to determine maximum mycelial growth) were replicated three times for each isolate in each generation.

**Fig 1:**
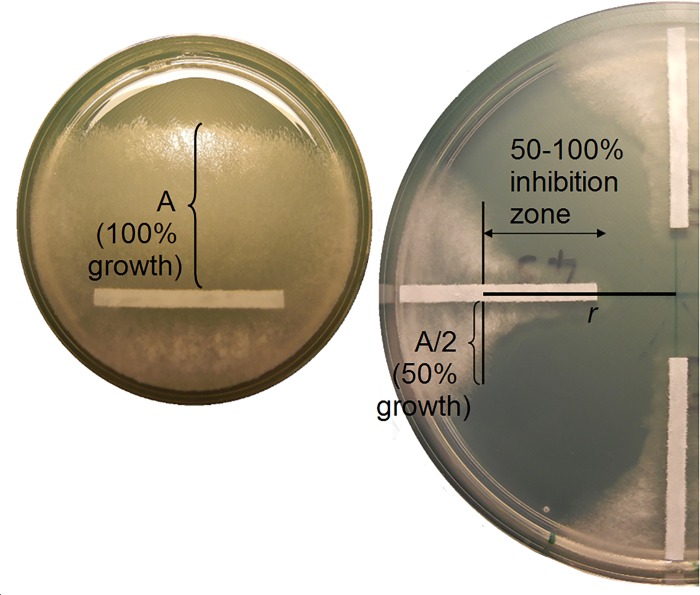
Method for identification of 50–100% growth inhibition zone of *Sclerotinia sclerotiorum* isolates grown on a fungicide concentration gradient. Mycelial growth perpendicular to the pre-inoculated filter strip after 40 hours on three replicated control plates were used to determine 100% growth (A), where A/2 is equal to 50% growth. At the same time point, mycelial growth on the fungicide-deposited gradient equivalent to distance A/2 was identified in both directions perpendicular to the pre-inoculated filter strip. Mycelium was harvested from the 50–100% inhibition zone in this experiment. To calculate the fungicide concentration at the 50% growth inhibition (EC_50_), an average of the two radial distances at which 50% inhibition was observed perpendicular to the filter strips was input into the SGE software. This example in the photos shows *S*. *sclerotiorum* growth on boscalid fungicide deposited in a concentric concentration gradient of 9.05 to 0.05 ppm from center to periphery of plate, with EC_50_ estimated as 0.14 ppm.

Inoculated Petri plates were incubated for 38–42 hours at 25°C, following which, myclial growth was measured to identify 50–100% growth inhibition for each isolate grown on each fungicide-deposited plate. First, growth on each negative control plate was measured to determine maximal growth (0% inhibition) of an isolate, which was the maximum length of mycelial growth perpendicular to and measured from the edge of the filter strip (Fig 1). This distance was measured on each of three replicated negative control plates and averaged to yield maximal growth distance, which was subsequently used to calculate 50% growth for each isolate. Second, mycelium in the 50–100% inhibition zone in both directions from each filter strip (Fig 1) was harvested and transferred to microtubes (as described above) for the subsequent generation and fungicide exposure. Each of nine isolates was repeatedly exposed to fungicides for a total of 12 generations. Cultures from each generation were maintained at room temperature for 3 weeks to allow formation of sclerotia, which were placed into microtubes, and both plates and sclerotia were preserved at 4°C for long-term storage.

### Fungicide Sensitivity Estimates

Fungicide concentration for 50% growth inhibition (EC_50_) was estimated in each generation. As above, mycelial growth in both directions equivalent to 50% growth from each filter strip on fungicide-deposited plates was identified. An average of the two measurements was used to estimate the corresponding radial distance up to 0.5 mm accuracy and input into the Autoplate 5000 software (Spiral Gradient Endpoint, Spiral Biotech Inc., Norwood, MA) to determine the corresponding fungicide concentration for each filter strip. Data from two experiments were analyzed separately using a Student’s t-test to determine if EC_50_ values of G0 and G12 of each isolate exposed to different fungicides were significantly different at *P* = 0.05.

### Molecular Genetic Analysis

DNA was purified from mycelium of each isolate: before exposure (G0), after growth on each fungicide for 12 generations (TM, Bos, Ip, Az, Py), and after growth on PDA for 12 generations (Con-G12). A DNeasy Plant Mini Kit (Qiagen Inc., Valencia, CA) was used according to instructions with 50–100 mg mycelium harvested from actively growing cultures, yielding 300–1000 ng DNA, and stored at -20°C until later use. DNA from each isolate was subsequently subjected to SSR genotyping and isolates showing mutation were additionally subjected to AFLP genotyping. Control isolates from G0 and G12 from Exp1 were genotyped to assess spontaneous mutations at SSR and AFLP loci.

Six SSR markers for loci that were polymorphic and showed unambiguous repeat lengths were used in the present study: 6–2, 17–3, 20–3, 55–4, 110–4, and 114–4 [28]. Three loci were hexa-, tri-, and dinucleotide repeats (6–2, 17–3, 20–3) and three were tetranucleotide repeats (55–4, 110–4, 114–4). Polymerase chain reaction (PCR) was carried out as described previously [28] using FAM-labeled primers. Prior to fragment analysis, amplicons were resolved in a 1.5% agarose gel stained with ethidium bromide to ensure the product was within the expected size range. Following amplification, 1.5 μl of each PCR product was mixed with 11 μl Hi-Di formamide (Applied Biosystems, Warrington, UK) and 0.2 μl GeneScan™ 600 LIZ® as a size standard. The product mixture was denatured for 5 min at 95°C and rapidly chilled on ice before shipping to Ohio State University Plant-Microbe Genomics Facility for fragment analysis on a 3730 genetic analyzer (Applied Biosystems Inc., Foster City, CA). SSR genotyping was performed on all isolates of both experiments, with the exception of isolate 587, which was removed from the study since fungicide-exposed isolates died during the experiment. Raw data was processed using GeneMapper software version 4.1 (Applied Biosystems) and a genotype table of fragment sizes was exported for further analysis.

Prior to AFLP genotyping, a total of 12 primer pairs were evaluated using three *S*. *sclerotiorum* isolates (Con_462_G0, Bos_587, Ip_467), which allowed identification of three primers that produced more than eight clear fragments in an agarose gel. AFLP genotyping was carried out using non-methyl sensitive restriction enzymes, based on the method described previously [32], with the following modifications. Pre-amplification and selective amplification primers were ordered from Life Technologies Corporation (Grand Island, NY). AFLP® Core Reagent Kit (Invitrogen™, Carlsbad, California) was used for restriction digestion and ligation steps according to instructions. Approximately 250–400 ng of genomic DNA was digested with *Eco*RI and *Mse*I. Thereafter, digested products were ligated with *Eco*RI and *Mse*I double stranded (ds) adapters provided. After ligation, the reaction mixture was diluted ten-fold with sterile Tris-EDTA (TE) buffer and used for pre-amplification.

Pre-amplification was carried out with *Eco*RI (5′-GTAGACTGCGTACCAATTC-3′) and *Mse*I (5′-GACGATGAGTCCTGAGTAA-3′) primers that were compatible with the respective oligonucleotide adapters used in ligation. The pre-amplification mixture of 50 μl included 5 μl of diluted restriction-ligation reaction, 0.1125 μM each of *Eco*RI and *Mse*I primers, 1× Taq polymerase reaction buffer, 0.2 mM of each dNTP, and 1 U of Taq polymerase (Invitrogen™). PCR was performed using a Mastercycler®Pro thermocycler (Eppendorf, Hamburg, Germany) with the first cycle at 72°C for 2 min, then initial denaturation at 94°C for 2 min followed by 30 cycles of 30 s at 94°C, 1 min at 60°C, and 2 min at 72°C. PCR products were diluted fifteen-fold with TE buffer and used as template DNA for selective amplification with primer pairs consisting of *Eco*RI and *Mse*I adapter sequences having 2–3 selective nucleotides each (*Eco*RI + *AAC* and *Mse*I + *CA*, *Eco*RI + *AAC* and *Mse*I + *CC*, *Eco*RI + TG and *Mse*I + *CA*). All *Eco*RI selective primers were 5′ labeled with fluorescent dye 6-FAM for fragment analysis. Each selective PCR mixture of 20 μl included 5 μl of diluted pre-amplification product, 0.25 μM each of *Eco*RI and *Mse*I primers, 1× standard Taq polymerase reaction buffer, 0.2 mM each dNTP and 1 U of Taq polymerase (Invitrogen™). PCR was performed for 36 cycles with the following cycle profile. The first twelve cycles consisted of 30 s DNA denaturation at 94°C, 30 s annealing at 65°C (-0.7 C/cycle), and 1 min extension step at 72°C. Remaining cycles consisted of 30 s at 94°C, 30 s at 56°C, and 1 min at 72°C. A final extension step of 5 min at 72°C completed the reaction.

Following AFLP amplification, 1.5 μl of each PCR product was mixed with 11 μl Hi-Di formamide and 0.2 μl of GeneScan™ 600LIZ^®^ internal marker. Mixtures were denatured at 95° C for 5 min and placed on ice, following which, samples were sent to Ohio State University Plant-Microbe Genomics Facility for fragment analysis. Raw data was processed using GeneMapper and individual AFLP bands automatically scored as either absent (zero) or present (one), with a binary genotype table exported for further analysis. The 600LIZ^®^ internal marker was capable of accurately determining fragments in the range of 20–600 bp; fragments less than 50 bp were ignored.

### SSR Mutation Rate Calculation

A previous study estimated that an actively growing strain of *S*. *sclerotiorum* experiences 24 nuclear divisions in hyphal growth per day [23]. In the present study, it took approximately four days to complete each generation (growth on inoculated filter strips, followed by growth on treatment plate) and there were 12 such generations (G0 to G12) per experiment. Each isolate was estimated to undergo a total of ~1,200 nuclear divisions during each experiment (24 nuclear divisions per day x 4 d x 12 generations). This value was used for estimates of mutation rates. First, the number of stepwise mutations at each locus was determined. This was as the absolute value of the difference in the length of the allele between G12 and G0 (non-exposed), divided by the length of the SSR repeat motif (ie. trinucleotide repeat = 3). This was then divided by 1,200 nuclear divisions, resulting in an estimate of the observed mutation rate at each locus for each isolate in each treatment.

Normal mutation rates for each of the *S*. *sclerotiorum* genotyped loci are not known, so we used those estimated previously from *Saccharomyces cerevisiae* [39]. The average mutation rate for SSR loci with 20–24 tandem di- tri- and tetranucleotide repeats was 1.3 x 10^−5^ mutations per locus per nuclear division. This rate was used in the current study to estimate the expected mutation rate of each locus and calculate fold-change in observed mutation rates compared to the expected mutation rates.

### AFLP Data Analysis

The binary AFLP genotype table produced by GeneMapper was used to generate a neighbor-joining (NJ) tree with Rogers index [40] since it gave the best separation between control and fungicide-exposed isolates. The *aboot* function of the R package ‘poppr’ version 2.0.2 [41] was used for tree construction, with bootstrap values determined by resampling 1000 times. The Con_594_G12 was not included in the above analysis since it had an overabundance of stutter peaks, resulting in the highest number of alleles (n = 248) among all control isolates (100 alleles more than G0). Isolate Con_594_G12 was used for clone-censored data because the process censoring (described below) removed spurious/variable alleles.

Similarities and dissimilarities of AFLP genotypes of control and treated isolates were analyzed using principal coordinate analysis (PCoA) [42]. This was performed using Adegenet R package version 2.0.0 [43]. PCoA uses eigenvalues derived from a distance matrix (or any measure of association) and produces a graphic in a low-dimensional Euclidian space. Analysis was conducted with several similarity coefficients available with the Adegenet package, where ‘simple matching index’ [44] was selected since it gave the best separation of isolates. Significant difference between clusters formed was tested with analysis of molecular variance (AMOVA) using GenAlEx version 6.5 [45]. Phi_PT_ is a haploid analogue of Wright’s F_ST_, where a value of 0.05 or less is generally interpreted to mean differentiation between populations is weak [46,47].

Congruence of results was evaluated using a discriminant analysis of principal components (DAPC) on AFLP data using Adegenet. DAPC is a multivariate method designed to identify and describe clusters of genetically related individuals that maximizes separation between groups while minimizing variation within group [48]. This method is also capable determining probability of membership for each individual to a predefined group (ex. control and fungicide-exposed). First, the *find*.*clusters* function was used to identify the optimal number of clusters. This was achieved by running *k*-means clustering algorithm for increasing number of groups and identifying the group number that maximized variation between groups. The *assignplot* was used to visualize membership probabilities (posterior values provided by discriminant analysis) after successful reassignment of individuals based on discriminant functions. Large probability values indicate defined population clusters, while low values suggest admixed groups.

### Censored Data

Control isolates at the start of the experiment (G0) and at the end of the experiment (G12) were used to assess spontaneous mutations at SSR and AFLP loci. For example, AFLP data of control isolates from G0 and G12 showed some polymorphic alleles attributed as probable random mutations that generated background noise in aforementioned analyses. Consequently, a second, censored data set was generated where these polymorphic alleles were removed, such that each control isolate from G0 and G12 had the same multilocus genotype (i.e. clones) and, to avoid redundancy, each was reduced to a single, representative genotype in data analysis. Since these alleles were observed to be polymorphic within the control lines, the corresponding alleles of fungicide-challenged isolates were also removed from their respective genotypes. This censored AFLP binary table was analyzed to construct a neighbor-joining tree and DAPC clusters as described above.

## Results

### Fungicide Exposure and Sensitivity

**Fig 2:**
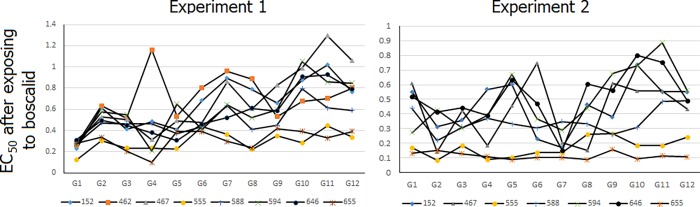
Change in EC_50_ values of *S*. *sclerotiorum* isolates exposed to sublethal doses of boscalid over 12 generations for experiment 1 and 2; G1 to G12 correspond to each generation.

Nine fungicide-sensitive *S*. *sclerotiorum* isolates were used in this study (ID 152, 462, 467, 555, 587, 588, 594, 646, and 655; Table 1). Since isolate 587 died early in experiment 1, there were a total of 104 isolates at the end of this study, which consisted of eight G0, 16 G12, and 80 fungicide-exposed from both experiments. Fungicide sensitivity (EC_50_) was estimated at each generation of the experiment, where results showed lack of a sustained trend throughout the course of the two experiments. For example, the estimated EC_50_ to boscalid during the course of the experiments is shown in Fig 2.

**Fig 3:**
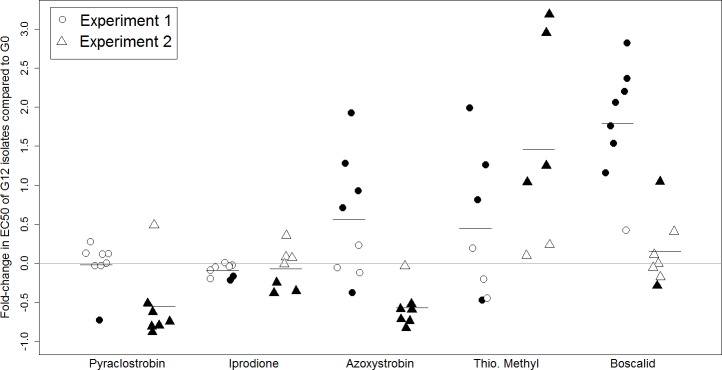
Change in fungicide sensitivity (EC_50_) of eight isolates after twelve generations of growth on a fungicide gradient of the respective commercial fungicide: pyraclostrobin, iprodione, azoxystrobin, thiophanate methyl, and boscalid. EC_50_ estimates were determined as the average of four replicates, where each point above represents the relative fold change in EC_50_ on an individual isolate basis, calculated as difference in EC_50_ at G12 and G0, divided by the EC_50_ at G0. Circles correspond with values from experiment 1 and triangles correspond to experiment 2, where solid filled shapes indicate EC_50_ values of G12 that were significantly different to G0, determined using a Student’s t-test (*P* ≤ 0.05).

Comparison of EC_50_ of each boscalid-exposed isolate at the end of each experiment to the corresponding negative control isolate showed all Exp1 isolates, with the exception of isolate 655, had a significantly higher EC_50_ (*P* ≤ 0.05; S1 Table). On average, boscalid-exposed isolates had a 1.788 fold-change in EC_50_ (Fig 3). Data from experiment 2 showed isolate Bos_594 had a significantly higher EC_50_ and Bos_467 had a significantly lower EC_50_ compared to their respective negative control isolates. Other boscalid-exposed isolates did not show a significant change from G0 (*P* > 0.05; $\bar{x}$ = 0.150 fold-change). Among the other fungicides in the first experiment, most isolates (n = 7/8) exposed to pyraclostrobin did not show a difference in sensitivity (*P* > 0.05; $\bar{x}$ = -0.020 fold-change), whereas azoxystrobin induced an upward shift in EC_50_ for four isolates ($\bar{x}$ = 0.565 fold-change; Fig 3 and S1 Table). In experiment 2 there was increased sensitivity for most isolates exposed to pyraclostrobin (n = 6/7; $\bar{x}$ = -0.553 fold-change) and azoxystrobin (n = 6/7; $\bar{x}$ = -0.572 fold-change). In both experiments, no isolates exposed to iprodione resulted in an increase in EC_50_ (${\bar{x}}_{Exp1}$ = -0.092, ${\bar{x}}_{Exp2}$ = -0.068); five resulted in higher sensitivity (Fig 3 and S1 Table). Thiophanate methyl-exposed isolate 152 and 646 had a higher EC_50_ in both experiments, while isolate 467 had a lower EC_50_ for experiment 1 (S1 Table); overall fold-change in EC_50_ for isolates were higher (${\bar{x}}_{Exp1}$ = 0.447, ${\bar{x}}_{Exp2}$ = 1.461).

### SSR Analysis

Each fungicide-exposed isolate (80) from both experiments, corresponding progenitor isolate at G0 (8) and corresponding non-exposed control isolate at G12 from experiment 1 were genotyped at six SSR loci (672 genotyping reactions). Isolate 655 of Experiment 1 exposed to thiophanate methyl (TM_655_Exp1) generated a high level of background noise at all six loci and was therefore removed from the analysis. Isolate 462 of experiment 2 was also removed from the analysis due to poor quality amplification. Additionally, isolate 588 exposed to thiophanate methyl (TM_588_Exp2) for all six loci showed poor amplification and were therefore not included in the analysis. The final analysis was based on 234 alleles from experiment 1 and 204 alleles from experiment 2, and control alleles for G0 and G12.

Multilocus genotypes of each isolate at G0 were compared to their corresponding G12 isolate genotype and showed no difference after 12 generations of growth on non-fungicide-amended PDA. Among all genotypes of fungicide-exposed isolates, however, showed 21 of 438 alleles were different than the expected progenitor (pre-exposure) genotype. These 21 mutations were present in 11 of the 80 fungicide-exposed isolates genotyped. All mutations followed a stepwise model of addition or removal of repeat units corresponding to the SSR repeat motif, with the exception of locus 6–2, which had 2 mutations, one with a 4 bp insertion and another with a 10 bp deletion. Among all other loci, insertions and deletions ranged from 4 bp and up to 80 bp, wherein the majority of mutations were less than 20 bp in length. Large deletions and insertions were observed at locus 114–4, which had a tetranucleotide repeat unit.

Collectively, mutations were comprised of 42 stepwise insertions and 85 stepwise deletions (Table 3). Most of the stepwise deletions were generated in iprodione- and azoxystrobin-exposed isolates (n = 40/85 each). Azoxystrobin-exposed isolate 646 in experiment 1 showed two deletions, one with 9-steps and the other with 5-steps at the locus 55–4 (Table 3). The largest total number of stepwise mutations (considering both insertions and deletions) was observed among isolates exposed to iprodione (44) and azoxystrobin (44), and the smallest total number of stepwise mutations was observed for boscalid-exposed isolates (6).

**Table 3:** Stepwise mutations at six microsatellite loci observed in eight *Sclerotinia sclerotiorum* isolates independently exposed for 12 generations to sublethal doses (50–100% inhibition) of five fungicides.

Fungicide	Exp. 1	Exp. 2	Total	Loci	Isolates	Per isolate
Boscalid	6	0	6	3	3	1–4
Insertions	4	0	4	-	-	-
Deletions	2	0	2	-	-	-
Iprodione	17	27	44	5	2	1–20
Insertions	4	0	4	-	-	-
Deletions	13	27	40	-	-	-
Thiophanate methyl	9^a^	10	19	5	3	1–6
Insertions	6	10^b^	16	-	-	-
Deletions	3	0	3	-	-	-
Azoxystrobin	17^c^	27	44	3	2	4–20
Insertions	4	0	4	-	-	-
Deletions	13	27	40	-	-	-
Pyraclostrobin	14	0	14	2	2	4–10
Insertions	14	0	14	-	-	-
Deletions	0	0	0	-	-	-

Number of mutations from experiment 1 and 2 as well as the nature of mutations in terms of deletion or insertion are given. Non-exposed control isolates showed no mutation in G12 compared to G0.There were 8 isolates per fungicide in Exp 1 and 7 isolates per fungicide genotyped in Exp 2, unless otherwise noted.

^a^ Thiophanate methyl had seven isolates.

^b^ Thiophanate methyl had six isolates.

^c^ One locus in isolate 646 generated a mixture of two genotypes, one a 4-step deletion and one a 9-step deletion; both are included in this estimate.

Isolate 594 expressed the highest number of stepwise mutations (78) while the lowest (6) was observed in isolate 462. In fact, isolate 594 expressed the highest number of stepwise mutations for both experiments (n = 24 and 54, respectively for experiment 1 and 2). In experiment 1, the following loci of isolate 594 showed mutations: boscalid-exposed locus 6–2; thiophanate methyl- and pyraclostrobin-exposed locus 17–3, iprodione-exposed loci 55–4 and 114–4. In experiment 2, mutations of isolate 594 were observed at loci 17–3 and 114–4 that were exposed to iprodione and azoxystrobin. Locus 20–3 did not yield mutations in either experiment, while the average number of stepwise mutations for all other loci ranged from 1.0 to 34.0. Isolates 152, 467, and 555 did not show mutations in any loci in either experiment, and no mutations in isolate 655 of experiment 1. The only isolates that had SSR mutations in experiment 2 were isolates 594 and 655.

SSR genotyping showed all fungicides induced mutations for each locus of the fungicide-exposed isolates, except at locus 20–3 that did not yield mutation. Locus 114–4 recorded the highest cumulative number of stepwise mutations (n = 68) while loci 6–2 and 110–4 resulted the lowest cumulative number of mutations (n = 2) at the end of both experiments. Therefore, among all fungicide treatments, the average number of mutations per isolate ranged from 0.026 to 0.906 per locus. Using known SSR mutation rates estimated for *S*. *cerevisiae* [39], it was expected that there would be a random mutation at a single locus after 76,923 nuclear divisions. Isolates in the present study underwent an estimated 1,200 nuclear divisions; therefore, the expected number of random mutations was 0.0156 per locus (1,200/76,923). In comparison, loci that mutated during fungicide-exposure in the present study had a mutation rate 1.7- to 60-fold higher than expected and, on average, 22-fold higher. Among the five fungicide-exposed isolates with mutations, the mutation rate per locus (excluding non-mutated loci) was 34-fold higher than expected under neutral conditions; one isolate (594) had an average 104-fold increase in mutation rate in both experiments.

### AFLP Analysis

A total of 33 *S*. *sclerotiorum* isolates were selected for AFLP genotyping, which included the 7 of the 12 isolates that had a SSR mutation in the fungicide-exposed treatment. Specifically, there were 16 isolates not exposed to fungicides (G0 and G12 from experiment 1) and 17 isolates that were exposed to fungicides. Among fungicide-exposed isolates, there were 10 TM-exposed isolates that originated from isolates 152, 555, 646 (two isolates each; from Exp1 and Exp2), 462, 588 (from Exp1), 467, and 655 (from Exp2), wherein isolates 462 and 655 exhibited SSR mutations. Also included were seven isolates from experiment 1 exposed to four fungicides (Az_646, Bos_646, Bos_555, Ip_467, Ip_594, Py_594 and Py_588,), among which, 5 of those isolates (Bos_646, Az_646, Ip_594, Py_594, and Py_588) had SSR mutations.

**Fig 4:**
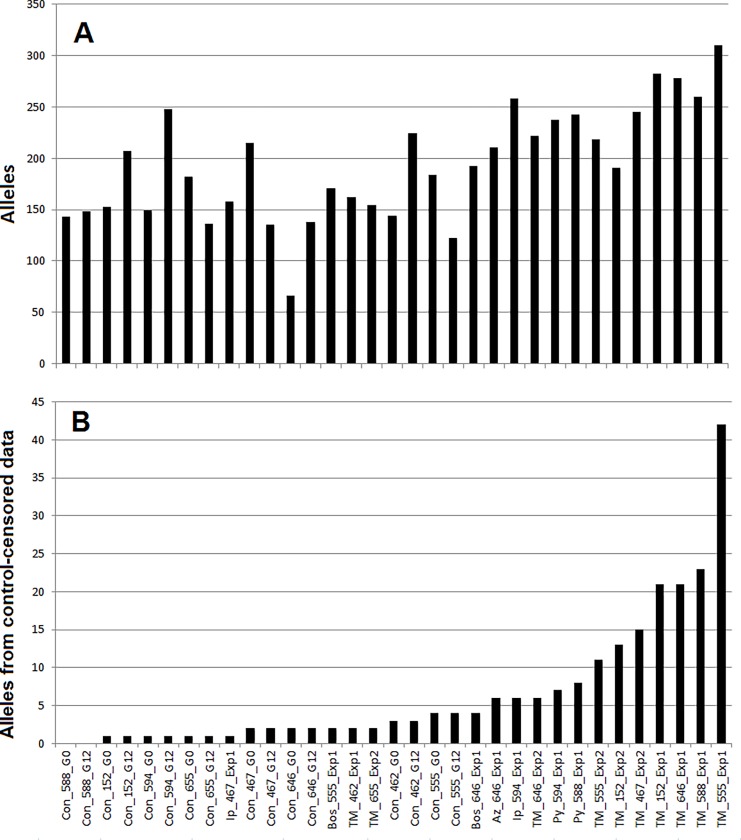
Number of AFLP alleles detected in *Sclerotinia sclerotiorum* isolates exposed to sublethal doses of fungicides as well as non-exposed controls. Isolates were exposed to fungicides for 12 generations before molecular characterization. Control isolates were characterized at the beginning (G0) of the experiment as well as after transferring for 12 generations (G12) on PDA without fungicide. Fungicides included boscalid (Bos), iprodione (Ip), thiophanate methyl (TM), azoxystrobin (Az), and pyraclostrobin (Py). Naming convention for each fungicide-exposed isolate is the fungicide used, isolate identification number, and experiment number; the experiment was repeated. All control isolates were from the first experiment and depicted as ‘Con’ followed by isolate name and generation (G0 or G12). **4A**) Original number of alleles detected for each isolate. **4B**) Number of alleles present after censoring AFLP data by removing loci polymorphic from G0 to G12 in the control for each isolate, resulting in the same multilocus genotype for the non-exposed control of each isolate.

**Fig 5:**
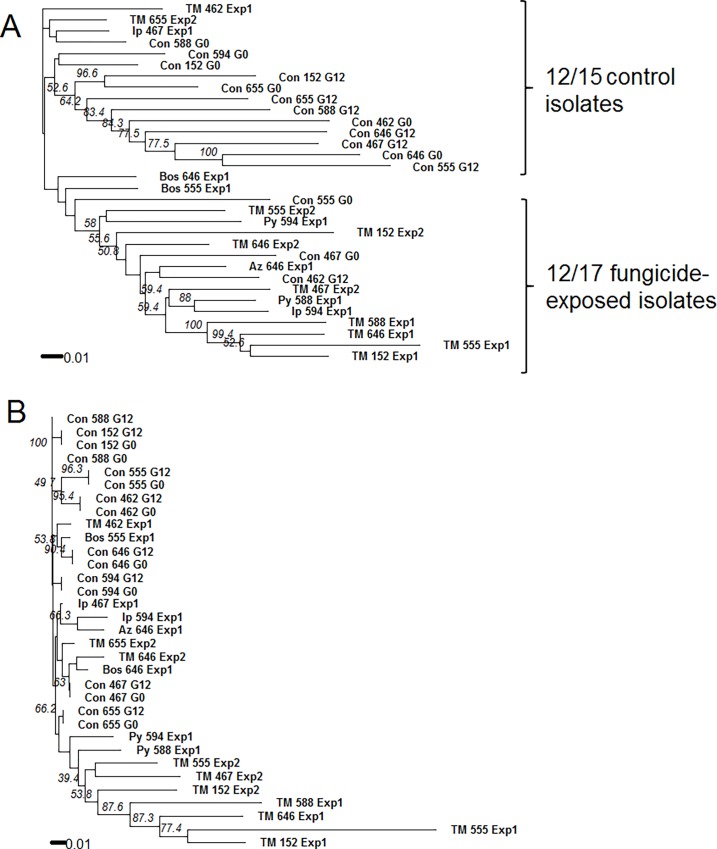
Neighbor joining (NJ) tree constructed from AFLP data of *Sclerotinia sclerotiorum* isolates exposed to sublethal doses of fungicides and non-exposed controls. Roger’s similarity coefficient was used to calculate pairwise distances between isolates. Isolates prior to fungicide-exposure at the beginning (G0) of the experiment and after fungicide exposure for 12 generations (G12) were genotyped using AFLP markers. Non-exposed, negative control isolates were simultaneously grown for 12 generations and included in the analysis; the experiment was repeated. Eight isolates were independently exposed to four fungicides: boscalid (Bos), iprodione (Ip), thiophanate methyl (TM), azoxystrobin (Az), and pyraclostrobin (Py). Fungicide used, isolate identification number, and experiment number are given for each taxon. Control isolates are depicted as Con followed by isolates name and their generation (either G0 or G12). All control isolates were used from the first experiment. Bootstrap values above 50% are shown at the beginning of clusters. **A**) NJ tree for uncensored data. **B**) NJ tree constructed from censored AFLP data, where loci polymorphic from G0 and G12 in control isolates were removed and corresponding loci of fungicide-exposed isolates also removed (see Fig 4).

Three AFLP primers resulted in a total of 602 polymorphic alleles among all fungicide-exposed and negative control isolates in this study; on average control isolates had 162 alleles/isolate, while fungicide-exposed isolates had an observed 223 alleles/isolate (Fig 4). There were no clonal genotypes. Genetic distance was calculated using Roger’s similarity coefficient and used to construct a NJ tree with two clusters that separated most fungicide-exposed and control isolates (Fig 5). The first cluster had a bootstrap value of 58% and contained 12 of 17 fungicide-exposed isolates and two control isolates (Con_462_G12 and Con_467_G0), and the second cluster contained 9 of 15 control isolates with bootstrap value of 52.6%.

**Fig 6:**
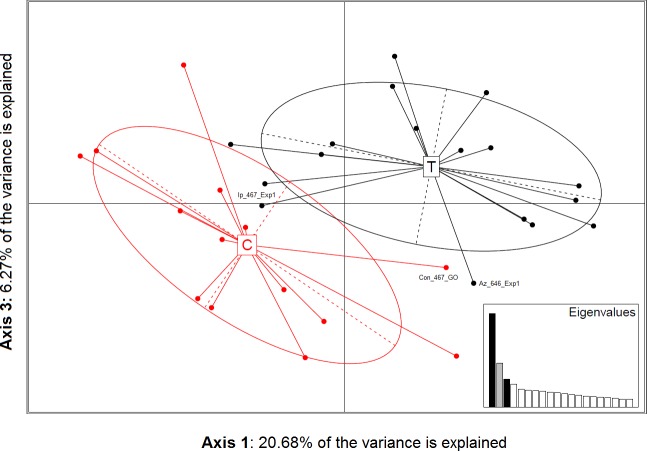
Principal coordinate analysis (PCoA) of AFLP fragments of *Sclerotinia sclerotiorum* isolates before and after exposure to sublethal doses of fungicides. Control isolates (both G0 and G12) are shown with a ‘C’ and fungicide-exposed isolates are denoted ‘T’ at the center of the respective groups, with each circle representing 95% of the variation associated with each group. Isolates Ip_467_Exp1, Az_646_Exp1, and Con_467_G0 have less than 90% membership probability to their respective treatment group; also shown in Fig 7.

Results of the PCoA showed the first three dimensions explained 37.64% of variation in the AFLP data, where each dimension represented 20.68%, 9.71%, and 6.27% of variation, respectively. The scatter plot of eigenvalues for axes one and three better separated fungicide-exposed and control isolates (Fig 6) than other combinations in two-dimensional space. PCoA results corroborated the topology of NJ tree of uncensored data (Fig 5). Fungicide-exposed and control groups were found to be significantly different with AMOVA (*Phi*_*PT*_ = 0.15; *P* = 0.001).

**Fig 7:**
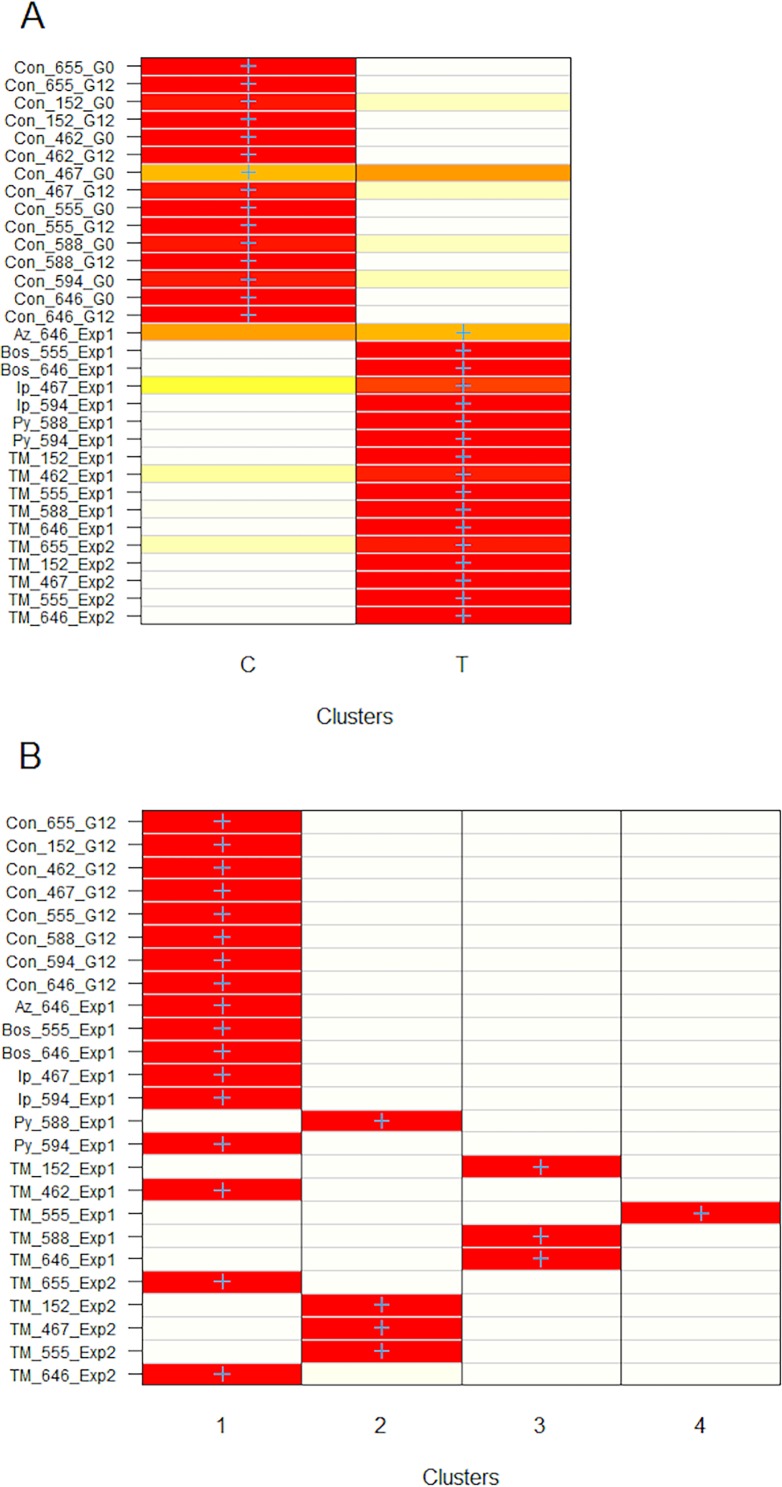
Heatmap of membership probabilities of *Sclerotinia sclerotiorum* isolates belonging to fungicide-exposed and non-exposed control clusters as shown in Fig 6. Probability of 100% is shown in red and 0% probability is white. Blue crosses represent the prior cluster assignment for isolates. **4A**) Membership of isolates pre-assigned to fungicide-exposed and non-exposed control groups, where isolates Az_646_Exp1, Con_467_G0, and Ip_467 had membership probability <90% to their respective group. **4B**) Membership probabilities of isolates after censoring AFLP data by removing loci polymorphic from G0 to G12 in the control isolates to result in the same multilocus genotype for each non-exposed control isolate; all isolates show 100% membership probability.

Discriminant analysis (DAPC) indicated two and three clusters as the optimal number of groups. Membership probabilities for assignment of each sample into two groups were congruent in 29 of 32 cases with isolates being assigned to either fungicide-exposed or non-exposed control groups (Fig 7A). Only three isolates had less than 90% probability of membership in their respective treatment group: Az_646_Exp1, Ip_467_Exp1, and Con_467_G0. When isolates were assigned to three clusters, 10 fungicide-exposed isolates and two control isolates grouped in one cluster while seven fungicide-exposed and control isolates each grouped in a separate cluster and a third cluster had six control isolates (*results not shown*).

It was expected that some alleles within the control isolates would vary from G0 to G12 due to the genome-wide, restriction enzyme based method of AFLP amplification. A second data set was constructed with alleles that varied within each control G0 to G12 were censored for the entire data set. The resultant data set consisted of eight controls (G0 and G12 represented by a single genotype) and 17 fungicide-exposed isolates. After loci in isolates were censored as explained above, the data contained 95 cumulative polymorphic alleles across all isolates, with an average of 2 alleles per control isolate and 11 per fungicide-exposed isolate (Fig 4). Seven out of 10 thiophanate methyl exposed isolates had more alleles than other fungicide-exposed isolates. Thiophanate methyl exposed isolates had an average of 16 alleles as compared to 5 for all other fungicide-exposed isolates.

There were 95 cumulative polymorphic alleles in the censored data used to construct the NJ tree (Fig 5B). The majority of isolates (n = 7/10) exposed to thiophanate methyl clustered together and away from control isolates, with a bootstrap value of 40%. Discriminate analysis did not identify a single solution for the optimal number of clusters and instead resulted in a range of 5 to 10 clusters as the optimal. Therefore, isolates were grouped into increasing number of clusters, starting at 3, and membership probabilities estimated. Using four clusters resulted in 100% membership probability for each isolate and was retained as the best fit (Fig 7B). Cluster 1 was the largest and contained all control isolates and nine fungicide-exposed isolates. The other three clusters contained 7 of 10 thiophanate methyl-exposed isolates, zero control isolates, and one pyraclostrobin-exposed isolate (Py_588_Exp1 in cluster 4). All fungicide-exposed isolates that clustered with control isolates in Fig 7B had fewer than eight alleles (Con_588_G0 to Py_594_Exp1 in Fig 4A).

Results from both experiments showed mutations are independent of isolates. For example, thiophanate methyl-exposed isolate 646 of Exp1 grouped outside of control isolates while the same isolate exposed to thiophanate methyl in Exp2 grouped with control isolates (Fig 7B). Similarly, thiophanate methyl-exposed isolates 152 and 555 of Exp1 and 2 clustered in different groups. Contributions of non-censored AFLP alleles that were most informative in separating control from fungicide-exposed isolates were identified. Nine of the 10 most informative alleles were generated by primer pair *Eco*RI + *AAC* and *Mse*I + *CA*. Alleles with greatest contributions were 24, 90, 115, 129 and 141 are the best candidate markers to test whether observed mutations were adaptive or random in future studies using *S*. *sclerotiorum* isolates. The range of these alleles observed in fungicide-exposed isolates varied from 16/17 to 11/ 17 and absent in 16/16 to 12/16 control isolates.

## Discussion

This experiment characterized the effects of sublethal fungicide stress on genetic variation, mutation rates, and fungicide sensitivity using *S*. *sclerotiorum* as a model system. This organism was selected because a previous study showed isolates grown continuously for one year on PDA had no mutations at SSR and AFLP loci, which was considered indicative of genomic stability [23]. Our results were similar and showed no variation at 6 SSR loci and no variation at 13 AFLP loci in isolates genotyped after 12 generations of growth on PDA (non-amended control). Fungicide exposure, however, generated SSR mutations and generated additional AFLP loci that were absent in the respective control isolates.

All observed SSR mutations in the present study were of fragment sizes consistent with that expected based on the locus repeat motif, suggesting mutations followed the stepwise mutation model and were a product of polymerase slippage during replication [30]. One exception was locus 6–2 that, according to [28], has a hexanucleotide repeat unit, yet termed a “polymorphic locus”, suggesting it may not be a true SSR locus. The number of stepwise mutations at each mutated locus was, in some cases, related to the number of repeats at the locus, which is consistent with previous studies that have shown mutation rates decrease for loci with fewer repeats [39,49]. For example, locus 114–4 had the most repeats (18) and yielded the greatest number of mutations in our present study, whereas locus 6–2 had the fewest repeats (5) and fewest mutations. In other cases there were SSR loci with many repeats and no observed mutations and vice-versa. For example, dinucleotide locus 20–3 contained 12 repeats and did not show mutation, whereas loci 17–3 and 110–4 (both with 9 repeats) that had 29 and 2 mutations. These results suggest that some SSR loci may be more vulnerable to mutation than others.

Variation at SSR loci was used to estimate the fold-change in mutation rates. Under normal conditions (without fungicide), we expected less than one mutation at each locus during the course of this experiment (0.0156 mutations per locus after 1,200 nuclear divisions). Our results showed mutated loci of fungicide-exposed isolates had an average 22-fold higher mutation rate. The number of tandem SSR repeats at the *S*. *sclerotiorum* loci used in our study ranged from 5 to 18 [28], which is considered typical of fungal genomes [39,50]. Our calculations, however, used the expected mutation rate for loci with 20–24 tandem repeat units, meaning fold-change in mutation rates we estimated for mutated loci of fungicide-exposed isolates in our study are likely underestimations. This is striking considering that one locus had a 60-fold higher mutation rate and one isolate (594) had an average 104-fold higher mutation rate in both experiments.

Our results corroborate findings of previous studies that showed different antifungal compounds induced mutations at SSR and ISSR loci in fungal pathogens *M*. *fructicola* and *A*. *alternata* [8–10]. Prolonged *in vitro* exposure of four *M*. *fructicola* isolates to sublethal doses of azoxystrobin fungicide yielded mutation at 8 of 15 SSR loci [10]. However, a DMI fungicide did not induce mutations at these loci. In a separate study, *B*. *cinerea* did not show SSR mutation in the presence of antifungal compounds pyrrolnitrin and iprodione [11]. It is interesting to point out that these three fungal species are closely related and belong to the same family, Sclerotinaceae. Despite the relative stability of the *S*. *sclerotiorum* genome and demonstrated stability of SSR loci, results of our study support findings of these previous studies showing SSR loci can mutate after exposure to sublethal fungicide stress *in vitro*.

One disadvantage to SSR genotyping is that these loci represent small fraction of the genome (typically less than 250 bp per locus), which is the reason AFLP genotyping was used as a complementary approach to survey mutations over a larger portion of the genome and assess different types of mutations. For example, AFLP mutations can occur when single-base mutations occur at the restriction site or at selective nucleotides immediately adjacent the site [51]. Such mutations result in loss of AFLP fragment amplification or may introduce a restriction site, thus generating AFLP fragments [51,52]. AFLP fragments may also vary in size due to insertions and deletions or other rearrangements within the amplified region.

AFLP genotyping showed fungicide-exposed isolates (12 of 17) accumulated mutations to group them as a separate group from control isolates in Neighbor-Joining analysis, with relatively high bootstrap support throughout the fungicide-exposed cluster. Two types of k-means clustering approaches (PCoA and DAPC) also supported fungicide-exposed isolates as a new and distinct group. This is significant because it was determined prior to initiating our experiment that the eight isolates used as progenitors for this study were genetically distinct [35]. Thus, results of NJ, PCoA, and DAPC analyses showed that the non-exposed isolates were more genetically similar to each other than they were to their fungicide-exposed counterparts. Furthermore, the variation that is detected by AFLP genotyping of fungicide-exposed isolates suggests that not all mutations were random. In the case of a DAPC, completely random mutations would lead to treatment groups that are unresolved from the progenitor genotypes, whereas our results showed clustering into two groups where membership probabilities correctly assigned 29 of 32 isolates with greater than 90% confidence into their respective treatment groups (fungicide-exposed or control) and was supported by AMOVA (*Phi*_*PT*_ = 0.15; *P* = 0.001). These results suggest mutations detected in AFLP genotyping are directionally driven by fungicide exposure.

Clone-censoring the AFLP data (removing alleles that were variable in negative control isolates) showed seven thiophanate methyl-exposed isolates retained the greatest number of alleles (Fig 4B), suggesting evidence that some mutations are fungicide-specific. For instance, a similar experiment conducted on *Monilinia fructicola* resulted in more TE movement (*Mfc1*) in isolates exposed to azoxystrobin than DMI fungicides, with no such TE movement observed in non-exposed controls [9]. This type of mutagen-specific mutation is not unexpected based on previous research in animal genetics that have shown mutagens yield both random (non-informative) and canonical mutations that are specific to the mutagen [53]. However, as shown in our AFLP data, mutagens that cause stress will yield a set of canonical mutations that are the same and a set that are unique canonical mutations, the genomic signature of a mutagen. Previous research on antifungals such as amphotericin B (azole group antifungal agent) and strobilurins showed these antifungals result in production of reactive oxygen species (ROS) downstream of their cellular targets [54–56]. ROS, such as hydroxyl radicals, damage DNA by formation of DNA strand breaks and modification of guanine bases in the pathogen genome [55,57–59]. Thus, it is likely that thiophanate-methyl specific mutations are detected in the present study due to the greater number of thiophanate-methyl-exposed isolates used in the AFLP analysis and it is likely other fungicide-specific mutations would have been detected if more isolates were genotyped using this approach. However, given the lack of sequence data corresponding to the AFLP fragments, further analysis should use whole genome sequencing.

Fungal pathogens exposed to stress may exhibit loss of heterozygocity (LOH) and aneuploidy [6,60], wherein both chromosomal changes and SNP mutagenesis can lead to acquired antimicrobial resistance. In our study, nearly all of the boscalid-exposed isolates in experiment 1 had a significantly higher tolerance at the end of the experiment compared to the negative controls (*P* ≤ 0.05). Approximately half of all isolates exposed to azoxystrobin and thiophanate methyl in the first experiment also had a significantly higher tolerance. Overall, however, there was not a clear trend of sublethal fungicide exposure leading to increased tolerance. To determine if presence of mutations was associated with fungicide sensitivity, regression analysis was performed but showed no significant association (*P*>0.05) between change in EC_50_ and mutation at either AFLP or SSR loci, suggesting many mutations were non-adaptive to growth with fungicide. However, these results do not rule out sublethal fungicide exposure as a mechanism for emergence of fungicide resistant phenotypes because our method selected mycelial growth that was 50–100% inhibited. Thus, we would not have selected mycelial growth exhibiting dramatic shifts in fungicide tolerance. We also did not subculture from these regions because such regions may have represented errors in fungicide deposition, which should necessarily be avoided. These results are a common feature of previous research on this topic using filamentous fungal plant pathogens [8–10].

As a survival mechanism, microorganisms react to damaged DNA by activating DNA damage repair pathways, however, this process may allow non-lethal mutations into the genome that may have unintended phenotypes. Although many mutations within genes would not have an obvious phenotype, some phenotypic changes were observed during our experimental study. For example, in both fungicide-exposed and control groups that exhibited slow growth and even death during the continuous transfer process. Isolate 587 was removed from the experiment since all treated and control isolates either died or experienced reduced and erratic growth by midway in the experiment, possibly due to expression of deleterious mutations. Isolates exposed to azoxystrobin and pyraclostrobin showed more aerial mycelia than control isolates, and isolates exposed to iprodione showed mycelial growth that was thinner than control isolates. Isolates exposed to boscalid produced sclerotia faster than control isolates with no other morphological changes, which is the opposite that was reported previously for boscalid-induced resistant isolates that lacked the ability to produce sclerotia [61].

Our results suggest that the majority of mutations observed in SSRs and AFLPs of *S*. *sclerotiorum* isolates were non-adaptive response to environmental stress, which is why regression analysis of the number of mutations, for either SSR or AFLP, with fold-change in EC_50_ did not have a significant relationship. When a population is under stress, including fungicide stress, mutations may serve as a mechanism for rapid adaptation to the environment, which may also introduce many random mutations not specific to adaptive resistance. Since only nine isolates were used in this study, it remains possible that a larger pool of isolates would result in fungicide-resistant isolates. In addition, our selection process did not target sections of mycelia with fungicide resistance by sectoring and slow shifts in fungicide sensitivity within portions of the mycelium may have been overshadowed once mixed with replicates in each generation. Indeed, when SSRs were characterized, isolate 646 gave two peaks for locus 55–4, which were distinct and high enough in amplitude to be considered true heterokaryotic or mixed-genotype peaks. To verify, these were re-amplified and results were the same.

Overall, results of this work conclusively demonstrate that sublethal fungicide exposure results in increased mutation rates in a fungus that is not known to evolve rapidly. Such stress may play a role under field conditions in generation of genotypic diversity in the *S*. *sclerotiorum* genome, which may be important for adaptation. In field conditions, large populations with more isolates may be affected by sublethal doses in the field through processes such as post-infection fungicide applications, elimination of early season sprays, increased application intervals, and low-rate fungicide applications. These processes may also play a more important role in the *S*. *sclerotiorum* pathosystems because primary infection by ascospores of senescing flowers are the target of fungicide applications, wherein mutations in the genome of a spore may play a more important role in pathogen evolution and resistance emergence. Indeed, such management practices have been speculated as a possible underlying mechanism of resistance emergence in field populations of the apple scab fungal pathogen *Venturia inaequalis* [13]. Results of the current study conclusively show *in vitro* sublethal fungicide stress induces mutations in the *S*. *sclerotiorum* genome, where future studies using whole-genome sequencing may shed more light on genomic damage specific to each class of fungicide and used to examine fungicide-resistant isolates for evidence of such mutagen exposure. Collectively, this information will be used to determine whether sublethal fungicide exposure plays a role in fungicide resistance or diversification in field populations of fungal plant pathogens, an important consideration for long-term sustainability of fungicides for disease control.

## Supporting Information

S1 Table Change in EC_50_ of *Sclerotinia sclerotiorum* isolates after exposure to sub-lethal doses of fungicide for 12 generations. (DOCX)
