# Supplementary material for: Effects of Sublethal Fungicides on Mutation Rates and Genomic Variation in Fungal Plant Pathogen, Sclerotinia sclerotiorum
Source: PLoS One. 2016 Dec 13;11(12):e0168079. doi: 10.1371/journal.pone.0168079 (PMC5154572; doi:10.1371/journal.pone.0168079)
Supplement: S1 Table — (DOCX) [file pone.0168079.s001.docx]

**Supplemental Table 1.** Change in EC_50_ of *Sclerotinia sclerotiorum* isolates^a^ after exposure to sub-lethal doses of fungicide for 12 generations

| **Experiment 1** | **Boscalid** | **Iprodione** | **Thio. Methyl** | **Azoxystrobin** | **Pyraclostrobin** |
| --- | --- | --- | --- | --- | --- |
| Increased | 152, 462, 467, 555, 588, 594, 646 | - | 152, 594, 646 | 462, 467, 646, 655 | - |
| No Change | 655 | 462, 467, 555, 588, 594, 646 | 462, 555, 588 | 152, 588, 594 | 462, 467, 555, 588, 594, 646, 655 |
| Decreased | - | 152, 655 | 467 | 555 | 152 |
| **Experiment 2** | **Boscalid** | **Iprodione** | **Thio. Methyl** | **Azoxystrobin** | **Pyraclostrobin** |
| Increased | 594 | - | 152, 467 646, 655 | - | - |
| No Change | 555, 588, 646, 655 | 588, 594, 646, 655 | 555, 594 | 594 | 555 |
| Decreased | 152, 467 | 152, 467, 555 | - | 152, 467, 555, 588, 646, 655 | 152, 467, 588, 594, 646, 655 |

^a^Isolate ID numbers listed here come from Table 1.
